# The juxtamembrane regions of human receptor tyrosine kinases exhibit conserved interaction sites with anionic lipids

**DOI:** 10.1038/srep09198

**Published:** 2015-03-17

**Authors:** George Hedger, Mark S. P. Sansom, Heidi Koldsø

**Affiliations:** 1 Department of Biochemistry, University of Oxford, South Parks Road, Oxford OX1 3QU, United Kingdom

## Abstract

Receptor tyrosine kinases (RTKs) play a critical role in diverse cellular processes and their activity is regulated by lipids in the surrounding membrane, including PIP_2_ (phosphatidylinositol-4,5-bisphosphate) in the inner leaflet, and GM3 (monosialodihexosylganglioside) in the outer leaflet. However, the precise details of the interactions at the molecular level remain to be fully characterised. Using a multiscale molecular dynamics simulation approach, we comprehensively characterise anionic lipid interactions with all 58 known human RTKs. Our results demonstrate that the juxtamembrane (JM) regions of RTKs are critical for inducing clustering of anionic lipids, including PIP_2_, both in simple asymmetric bilayers, and in more complex mixed membranes. Clustering is predominantly driven by interactions between a conserved cluster of basic residues within the first five positions of the JM region, and negatively charged lipid headgroups. This highlights a conserved interaction pattern shared across the human RTK family. In particular predominantly the N-terminal residues of the JM region are involved in the interactions with PIP_2_, whilst residues within the distal JM region exhibit comparatively less lipid specificity. Our results suggest that JM–lipid interactions play a key role in RTK structure and function, and more generally in the nanoscale organisation of receptor-containing cell membranes.

The structure and function of membrane proteins are influenced by their lipid environment. Receptor tyrosine kinases (RTKs) are ubiquitous receptors in mammalian cell membranes, which transduce information about cellular environment and interactions across the plasma membrane to complex intracellular signalling networks^1^. Human RTKs comprise a family of 58 members divided into 20 subfamilies^2^,^3^. The diverse cellular processes regulated by these proteins include cell growth and division, differentiation, metabolism, migration and apoptosis^4^,^5^. The importance of RTK function is illustrated by the array of diseases, including cancer, diabetes, bone disorders, atherosclerosis and inflammatory conditions, which have been linked to pathogenic mutations in RTKs and the signalling systems they control^6^,^7^. Several RTK inhibitors are currently in clinical trials. Therefore, understanding the structure and function of these receptors is of considerable biomedical importance^8^.

Almost all members of the human RTKs share a common molecular architecture consisting of an extracellular ligand binding ectodomain, a single transmembrane (TM) helix, and an intracellular region composed of a flexible juxtamembrane (JM) region followed by a protein kinase domain and a C-terminal region (see Fig. 1a).

Most RTKs can exist as monomers in an inactive state. However the insulin receptor (INSR) and insulin-like growth factor 1 receptor (IGF1R) exist as constitutive disulfide linked dimers^4^,^5^,^9^. Increasingly it is recognised that certain receptors, including the epidermal growth factor receptor (EGFR/ErbB1), can exist as inactive predimers and also in higher oligomeric states, while larger scale clustering within the membrane has also been observed^10^,^11^,^12^. It is well established that ligand binding to the extracellular ectodomains promotes receptor dimerization, and induces conformational reorganisation in existing dimers, leading to activation of protein kinase activity within the intracellular domains. The activated kinase domain of each monomer trans-autophosphorylates tyrosine residues in the C-terminal domain, JM region and/or activation loop of the opposing monomer leading to further downstream activation. RTK activity is regulated by multiple factors including interactions with membrane lipids such as GM3^13^, phosphorylation by downstream *trans*-factors in feedback loops, clathrin mediated receptor endocytosis *via* NPxY motifs^14^, ubiquitination, and by *cis*-elements including the kinase domain activation loop and the JM region^1^.

The JM region is a flexible region normally consisting of 40 or more residues. It links the C-terminus of the transmembrane helix to the intracellular protein kinase domain. Previous efforts to understand RTK structure and function have primarily been focused on the ligand binding and protein kinase domains. The JM region is generally absent from crystal structures as it is often unstructured and intrinsically flexible. Due to difficulties in obtaining high resolution structures of full length RTKs, a modular interdisciplinary approach to RTK structure determination has been employed, with the X-ray crystallographic and NMR structures of the individual domains of many RTKs now known. These approaches have been successful, and in the case of ErbB1 structural data on isolated parts has been assembled into models of the full length receptor *in silico*^15^,^16^,^17^. The full length models are illustrative of a more recent shift in focus towards understanding whole receptors including the JM region, rather than isolated components; a goal which is also being pursued utilizing CryoEM^18^.

The JM region is not simply an inert linker between the TM section and the kinase domain. It serves important regulatory roles and is essential for ErbB1 dimerization and activation of protein kinase activity^19^,^20^. Additionally the JM region often contains trafficking signal motifs^1^, and binding sites for calmodulin^21^. Autoinhibition of protein kinase activity by the JM regions has been reported for numerous RTKs, including Fms-like tyrosine kinase (FLT3)^22^, KIT^23^, Muscle-specific kinase (MuSK)^24^ and Ephrin (Eph) family RTKs^25^. It has been shown in most cases that the JM regions form inhibitory contacts with the kinase domain, which must be disrupted for receptor activation^9^ to occur. In contrast, however, the JM region of EGFR acts as an activation domain, with alanine scanning mutagenesis defining the C-terminal JM region as being essential for receptor activation. A crystal structure of the asymmetric dimer reveals that the JM regions form multiple stabilizing contacts with each other^26^. Whether autoinhibitory or acting as an activation domain, in all cases studied to date, the JM region is essential for normal receptor function.

To date, the interactions between JM regions and anionic lipids have been studied for ErbB1 using experimental^27^,^28^ and computational approaches^15^,^29^. PIP_2_ is an essential signalling lipid that is present in the inner leaflet of the plasma membrane. *In vitro* FRET labelling studies with ErbB1 JM peptides and phospholipid vesicles showed the JM construct was able to induce PIP_2_ clustering^30^. Surface plasmon resonance (SPR) studies demonstrated strong binding between PIP_2_ and the JM region of ErbB1^27^. PIP_2_-JM interactions have also been observed in TM-JM constructs of rat epidermal growth factor receptor 2 (ErbB2)^31^. This interaction is likely to be mediated *via* electrostatic interactions between a cluster of basic residues in the JM region and the multiple phosphoryl groups of the headgroup of PIP_2_, with interaction abolished in R/K > N neutralisation mutants^27^.

Clustering of the anionic lipid phosphatidylserine (PS) around the JM region has been observed in atomistic MD simulations of full-length ErbB1^15^, and specific interactions with PIP_2_ were recently observed in multiscale simulations^29^. Interactions of the JM region with PIP_2_ were favoured over PS interactions and a neutralising Asn mutant exhibited fewer interactions^29^, consistent with an ‘electrostatic engine model' for ErbB1 JM involvement in receptor activation^30^. In addition to these studies demonstrating PIP_2_/PS-JM interactions of ErbB1/2, manipulation of PIP_2_ levels by pharmacological knockdown of PIP_2_-metabolizing enzymes demonstrated that ErbB1 receptor activity is PIP_2_ dependent^27^, thus adding ErbB1 to the list of membrane proteins modulated by PIP_2_^32^,^33^.

The presence of basic residues within the cytoplasmic JM region of several signalling receptors has been suggested to play a role in protein-lipid interactions^27^,^34^. Indeed the presence of basic residues within the JM region has been shown to be an inherent feature of all single transmembrane spanning proteins within eukaryotes. This significant enrichment in basic residues is present in proteins both within the plasma membrane and the golgi, and to a lesser extend the ER^35^. Despite the importance of lipid interactions revealed by these studies, the interactions of receptors with the membrane lipids have in the past been somewhat neglected within the RTK field^1^ (as noted by e.g. Coskun *et al.*^13^). However, methodological advances and a growing body of literature demonstrating their critical nature has led to an appreciation of protein-lipid interactions as a key aspect of the biology of RTKs^36^.

In contrast to previous studies, which have been limited to a single species of receptor, here we explore the specific interactions of the JM regions of the entire family of human RTKs with the anionic lipids PS and PIP_2_. We initially sought to test the hypothesis that multiscale MD simulations can reveal TM-JM interactions of RTKs with anionic lipids by focussing on two systems: the INSR and EphA2 receptors. Based on these two test systems, we then created a high throughput approach to allow us to perform simulations of all 58 human RTKs, and test whether the interactions between the JM region and anionic lipids were conserved over this entire family of diverse receptors. This allows us to, for the first time, obtain insights into the specific nature of lipid interactions and their degree of conservation across the entire family of human RTKs.

## Results

**Figure 1:**
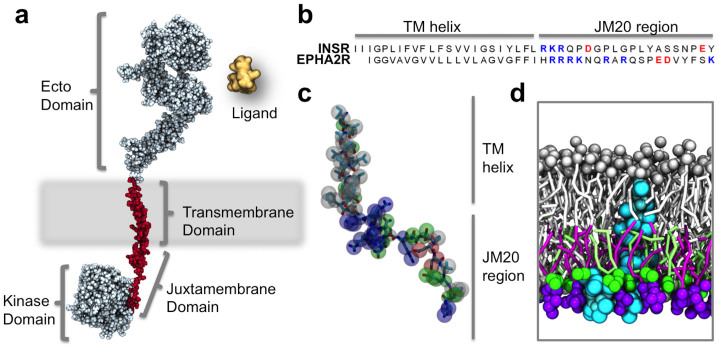
RTK architecture includes a JM region enriched in basic residues. (a) Schematic representation of a generic RTK model (generated using MODELLER^69^ based on PDB id: 2GS6^70^, 3GOP^26^, 2JIU^71^ 2M20^72^ and 3NJP^73^) with an extracellular ligand binding ectodomain, a single transmembrane spanning helix, a basic JM region, and a kinase domain. The TM and JM regions have been highlighted in red. (b) Sequence of INSR and the EphA2 receptor with charged residues within the JM region highlighted with basic residues in blue and anionic residues in red. (c) Protein model of the TM-JM region of the EphA2 receptor with CG beads overlaid onto an atomistic model: basic residues are shown in blue, acidic in red, polar in green, and non-polar in white. (d) Cross section of the same EphA2 receptor TM-JM model embedded in a lipid bilayer containing 20% PS and 20% PIP_2_ within the inner leaflet.

Models of the INSR and EphA2 receptors TM-JM regions consisting of the TM helix and first 20 residues of the JM region (Fig. 1b) were converted into coarse-grained (CG) representation (Fig. 1c) and inserted into phosphatidylcholine (PC) bilayers using self-assembly simulations^37^. Reduced representation methods such as CG^38^,^39^,^40^ and hybrid models^41^ have previously proven to be valuable approaches in the exploration of protein-lipid interactions. Asymmetric bilayers were constructed utilizing the method previously described in Koldsø *et al.*^42^. The asymmetric bilayers had a composition of 80:10:10 of PC:PS:PIP_2_ which closely mimics the *in vivo* anionic lipid composition of the inner (i.e cytoplasmic) leaflet (Fig. 1d). The simulations were run for 1 μs, with triplicate repeats performed for each system.

### JM lipid interactions

**Figure 2:**
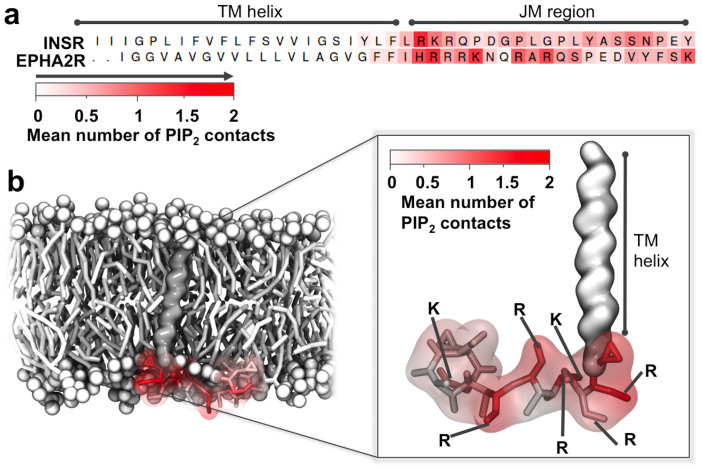
Interactions between PIP_2_ and the JM region. (a) TM helix-JM sequence of INSR and the EphA2 receptor coloured by the extent of PIP_2_ headgroup contacts from white (low) to red (high). (b) The CG structure of the EphA2 receptor TM-JM model with residues also coloured according to the extent of contacts with the PIP_2_ headgroup. Contacts were calculated over 3 × 1 μs repeat simulations using a 6 Å cutoff.

The JM region moved towards the bilayer during the simulation and oriented itself to lie along the membrane surface, thereby allowing the basic residues to form favourable interactions with the anionic lipid headgroups (Fig. 1d and 2b). In particular, we observed favorable interactions between the headgroups of PIP_2_ and the JM region of both the INSR and the EphA2 receptor as evident from the mapping of the mean number of interactions during the simulation onto the sequences of the proteins (Fig. 2a). Not surprisingly, PIP_2_ exhibited the highest frequency of contacts with Arg and Lys residues, an intermediate level of contact with polar residues, and a low level contact with acidic and hydrophobic residues (Fig. 2).

### Lipid clustering

**Figure 3:**
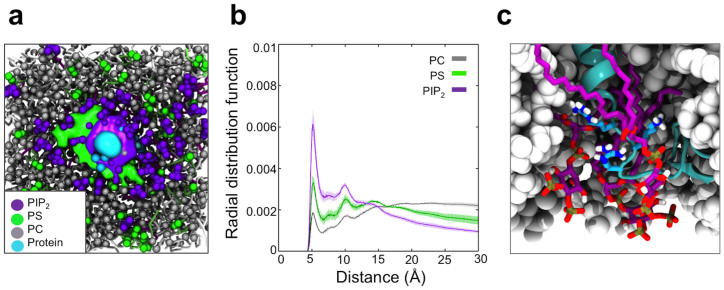
Lipids clustering around the JM region within the INSR system. (a) Occupancy density maps of the inner leaflet surface showing spatial occupancy for the phosphoryl group attached to the glycerol moiety of the PIP_2_ headgroup (purple), the PS headgroup (green), and the Arg and Lys residues of the JM region (cyan). Occupancy maps were computed from concatenated 3 × 1 μs simulations of each system, using Volmap in VMD. (b) Radial distribution functions (RDFs) for lipid headgroups of PIP_2_, PS, and PC with respect to INSR. The RDFs shown are the mean values from 3 × 1 μs simulation repeats, with the standard deviation displayed as error bars. The area under each RDF curve is normalized to unity to aid comparison between the different lipids species. (c) A cluster of PIP_2_ lipids around the INSR receptor JM region in an AT simulation. The protein has been shown in cyan with the basic juxtamembrane regions in sticks coloured according to atom type with carbon atoms in cyan. PIP_2_ lipids within 6 Å of the protein have been shown as sticks coloured according to atom type, with carbon atoms in purple. The PC and PS lipids are shown as gray spheres.

PIP_2_ was seen to form the overall highest degree of contact with JM residues, followed by PS, and then PC; as observed from the density maps for each lipid (Fig. 3 and Supplementary Fig. 1). Density maps of the inner membrane showing the spatial occupancy of each lipid headgroup over the course of the simulation were used to visualise lipid distributions within the INSR system (Fig. 3a). The EphA2 receptor system showed the same overall pattern (Supplementary Fig. 1). This analysis revealed clustering of lipids into ordered ring-like patterns around the protein. The mean number of PIP_2_ lipid headgroups within 6 Å of the protein (corresponding to the first interaction shell) was between 4 and 6 for both systems. No such clustering was observed in a control system with only the transmembrane helix of INSR and no JM region (Supplementary Fig. 2).

Spherical radial distribution functions (RDFs) were computed for each lipid species to explore lipid distributions with respect to the protein (Fig. 3b). For both systems, lipids were seen to be distributed into shells around the protein with RDF peaks at 5 Å, 10 Å and 15 Å, consistent with results for the gp130 JM region which was observed to induce similar patterns of PS clustering^34^. Within the INSR system, the magnitude of these peaks for PIP_2_ and PS decreased 5 Å > 10 Å > 15 Å, indicating decreasing probability of finding these lipids upon moving away from the protein (Fig. 3b). The same pattern was observed for the EphA2 receptor system (Supplementary Fig. 1b). The normalised RDF at 5 Å decreased when going from PIP_2_ to PS and further for PC, indicating PIP_2_ has the strongest preference for interaction with the JM region, followed by PS, and then PC. No such preference was observed in control simulations of the INSR TM helix without a JM region (Supplementary Fig. 2). In simulations with only PS:PC and with only PIP_2_:PC, the RDFs for PIP_2_ and PS were very similar at 5 Å (Supplementary Fig. 3), indicating similar degrees of clustering within the first interaction shell. This dramatic drop in RDF for PS in the presence of PIP_2_ suggests the more anionic PIP_2_ headgroup outcompetes PS for interaction with the JM region. Both of these observations are consistent with clustering of anionic lipids *via* electrostatic interactions with basic JM residues.

**Figure 4:**
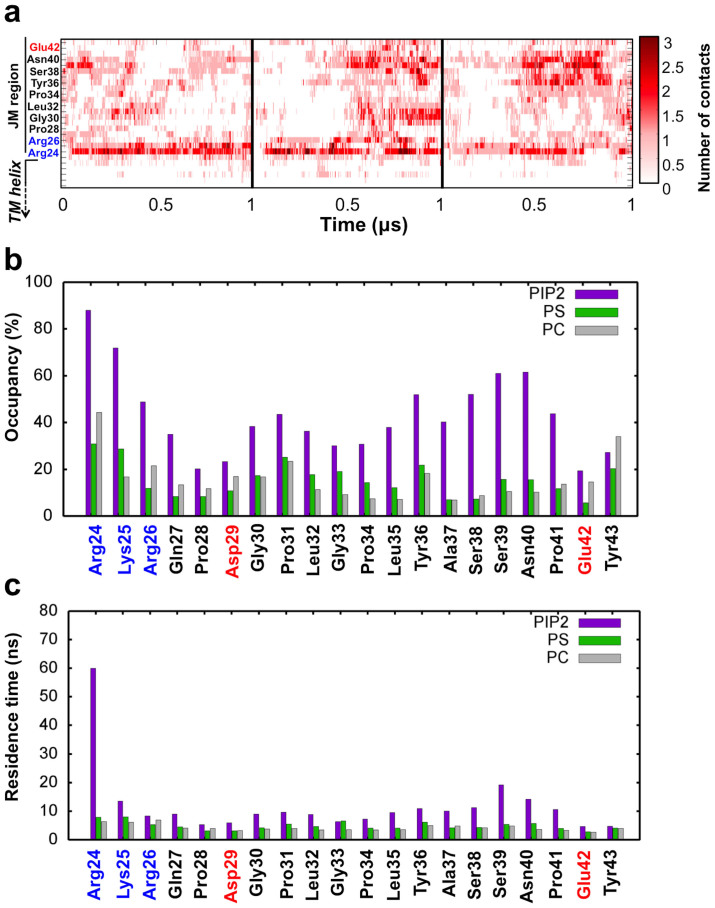
Dynamics of PIP_2_ interaction with the JM region. (a) Matrix showing the number of contacts per frame between each residue of the INSR JM region and the PIP_2_ lipid headgroup as a function of time. The time course shown is from concatenated 3 × 1 μs CG simulation repeats. A cutoff of 6 Å was used to define contact. (b) Percentage of simulation time at least one lipid is in contact with each residue within the JM region. PIP_2_, PS and PC are shown in purple, green and gray respectively. (c) Mean lipid residence time (occupancy time divided by number of association events) for concatenated 3 × 1 μs runs plotted for each JM residue.

To enable analysis of the dynamics of protein-lipid contacts, we also calculated the number of each lipid within 6 Å of each protein residue for each frame (Fig. 4). The results showed the number of contacts for each residue oscillates over the course of the simulations. This demonstrates that lipids do not bind irreversibly, but rather their association is highly dynamic such that they associate and dissociate with the JM region multiple times over the course of the simulation (Fig. 4a). Basic residues within the JM region were seen to form the most stable long-lived interactions with PIP_2_ and PS, whereas polar residues formed significantly more transient interactions with greater dynamic exchange with the surrounding lipid population. The percentage of time at least one of each lipid species was in contact with the JM region was also explored (Fig. 4b), which further supported the favourable interactions between PIP_2_ and basic residues revealed by our RDF and time-averaged contact data (Fig. 3). Based on the occupancy time and number of association events we additionally calculated a mean residence time between each lipid species and each residue of the JM region (Fig. 4c). This calculation clearly indicates that the interactions between basic residues in INSR (in particular Arg24) are long lived and stable compared to more transient interactions between polar residues and lipids. Again it was observed that the interactions with PIP_2_ over the other lipid species were very selective. A similar pattern was seen for the EphA2 receptor where all basic residues exhibit interactions with PIP_2_ for most of the simulation time; and the interactions between PIP_2_ and Lys26 were observed to be particular favourable (Supplementary Fig. 4). Additionally, we performed *in silico* mutagenesis in the EphA2 receptor in which all basic JM residues were mutated to leucine (Supplementary Fig. 5). This was chosen as leucine has a substantive hydrophobic side chain and thus differs from the basic residues mainly in terms of charge rather than size. In these simulations we observed loss of stable contacts at mutated positions and an overall decrease in the level of PIP_2_ and PS clustering. Some level of contact between lipids and the JM region was maintained due to interactions with polar residues and random lateral diffusion.

### AT simulations

To refine our CG observations, the final snapshots from 1 μs CG simulations of the INSR and EphA2 receptor systems were converted to AT detail and simulated for 50 ns of AT-MD. Root mean square fluctuations (RMSFs) were calculated for the Cα particle for both the CG and AT simulations, and used to compare the dynamic behaviour of each protein system (Supplementary Fig. 6). The RMSF values and profiles vs. residue number were very similar for the CG and AT simulations (Supplementary Fig. 6), indicating comparable degrees of JM flexibility at the two levels of resolution. Over the course of each 50 ns AT simulation the same patterns of lipid clustering were observed as in the CG simulations, with PIP_2_ having the greatest propensity to cluster and interact with the JM region (Fig. 3c and Supplementary Fig. 1c).

Analysis of lipid-protein contacts over time showed that the stable contacts of the PIP_2_ lipid headgroup with basic residues were maintained in the atomistic systems (Supplementary Fig. 7). Visual inspection of the trajectory also revealed that this contact occurred *via* both electrostatic interactions between the phosphoryl groups of the PIP_2_ headgroup and positively charged Lys and Arg side chains, as well as *via* some level of interaction of these residues with hydroxyl groups of the inositol moiety of PIP_2_. PS was seen to interact with basic side chains *via* both its phosphoryl and carboxylate moieties. Interestingly, a high level of contact was also seen at the C-terminus of the INSR JM region, which contained no basic residues. This contact may be a function of the multiple proline kinks found in the INSR JM region which appear to cause it to adopt a conformation in which the JM region is slightly bent upwards to sit comparatively deep in the membrane (Supplementary Fig. 8 and Fig. 3c). This observation demonstrates slight differences in how the JM regions of RTKs interact with the membrane surface, despite overall broad similarities.

The high similarity between the behaviour observed within the CG and the AT simulations suggest that the CG representation is sufficient to fully capture the overall pattern and dynamics of RTK TM-JM region interactions with anionic lipids.

### More complex membranes

Simple model bilayers provide useful test systems for exploring specific interactions. However the lipid composition of a mammalian plasma membrane is significantly more complex^43^,^44^. To better understand how this increased complexity may influence the interaction between anionic lipids and JM residues, we embedded both the INSR and the EphA2 receptors in bilayers of composition approximating that found in mammalian cell membranes^42^, mainly composed of PC and phosphatidylethanolamine (PE) but also including glycolipids (GM3) within the outer leaflet and anionic lipids within the inner leaflet (PS and PIP_2_), and performed 1 μs of CG simulation on these systems. The final composition within the outer leaflet was PC:PE:GM3:Cholesterol (50:15:10:25), while the inner leaflet had the following composition; PC:PE:PS:PIP_2_:Cholesterol (10:40:15:10:25), see Supplementary Fig. 9.

The RDFs for PIP_2_ and PS around the JM region indicated no significant deviation in their relative distribution from those observed in simple bilayers (Supplementary Fig. 10a+b). However the normalised probability decreased slightly. This qualitative decrease is likely to be a function of the reduced PIP_2_ and PS concentrations within the plasma membrane systems. The diffusion for all lipid types were also decreased, which is a general trend as the complexity and crowding of membranes increases^42^,^45^. Protein-lipid interaction plots (Supplementary Fig. 10c+d) showed good agreement with those for lipids in simple bilayers, with PIP_2_ forming the most stable interactions with basic JM residues and exhibiting the overall highest degree of contact. Our simulations of RTKs within more complex mixed lipid membranes further support our CG and AT findings from simple asymmetric membrane systems.

### All 58 human RTKs

**Figure 5:**
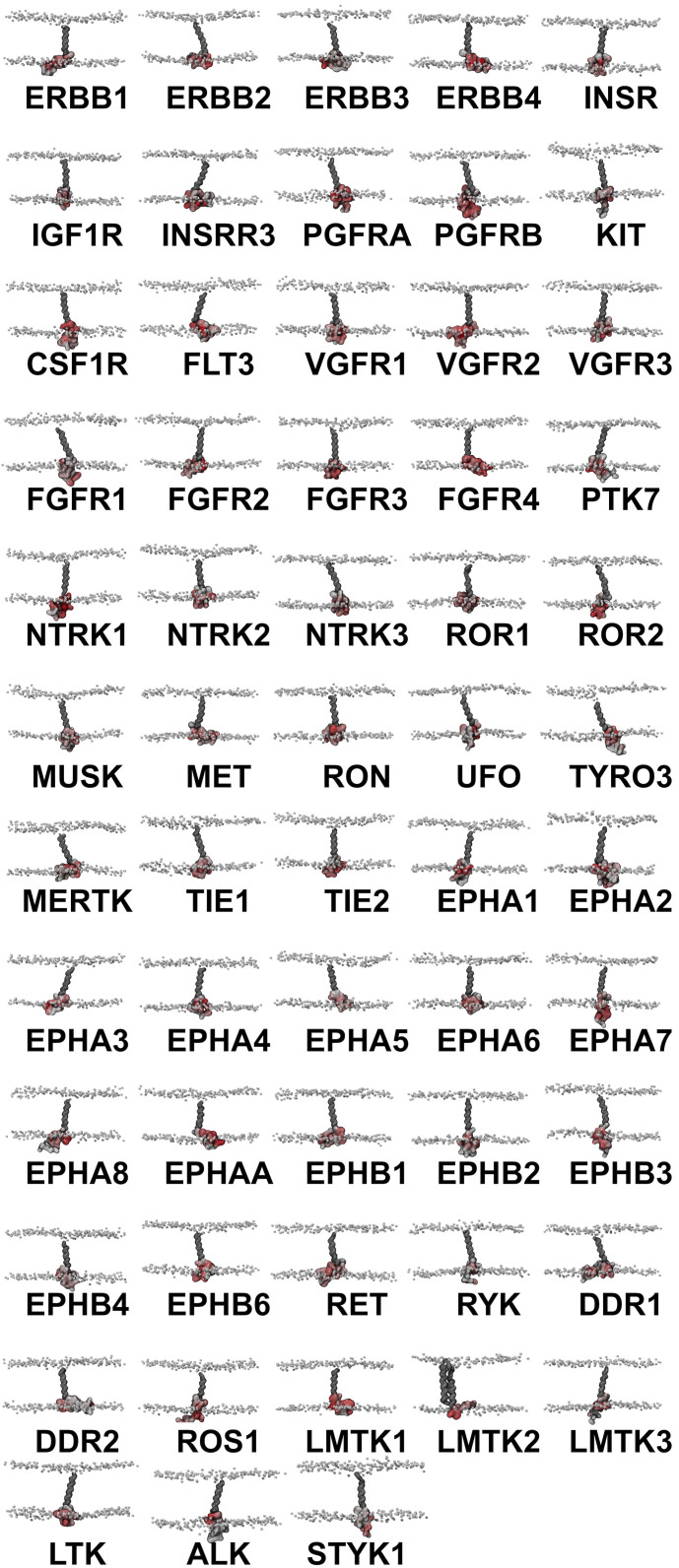
Simulations of all 58 human RTKs. All proteins were embedded into PC:PS:PIP_2_ (80:10:10) bilayers and simulated for 3 × 1 μs. The lipid headgroups are shown by white spheres and all the TM-JM models are coloured according to the extent of contacts with the PIP_2_ headgroup. Contacts were calculated over 3 × 1 μs repeat simulations using a 6 Å cutoff.

We extended our CG simulations to the TM-JM regions of all 58 human RTKs in order to evaluate the extent to which lipid interactions and clustering are conserved across the entire family. TM-JM models of each RTK were built and embedded in asymmetric PC:PS:PIP_2_ (80:10:10) bilayers using the protocol already described for the INSR and EphA2 receptors. Three runs of 1 μs simulations were performed for each RTK system (Fig. 5).

**Figure 6:**
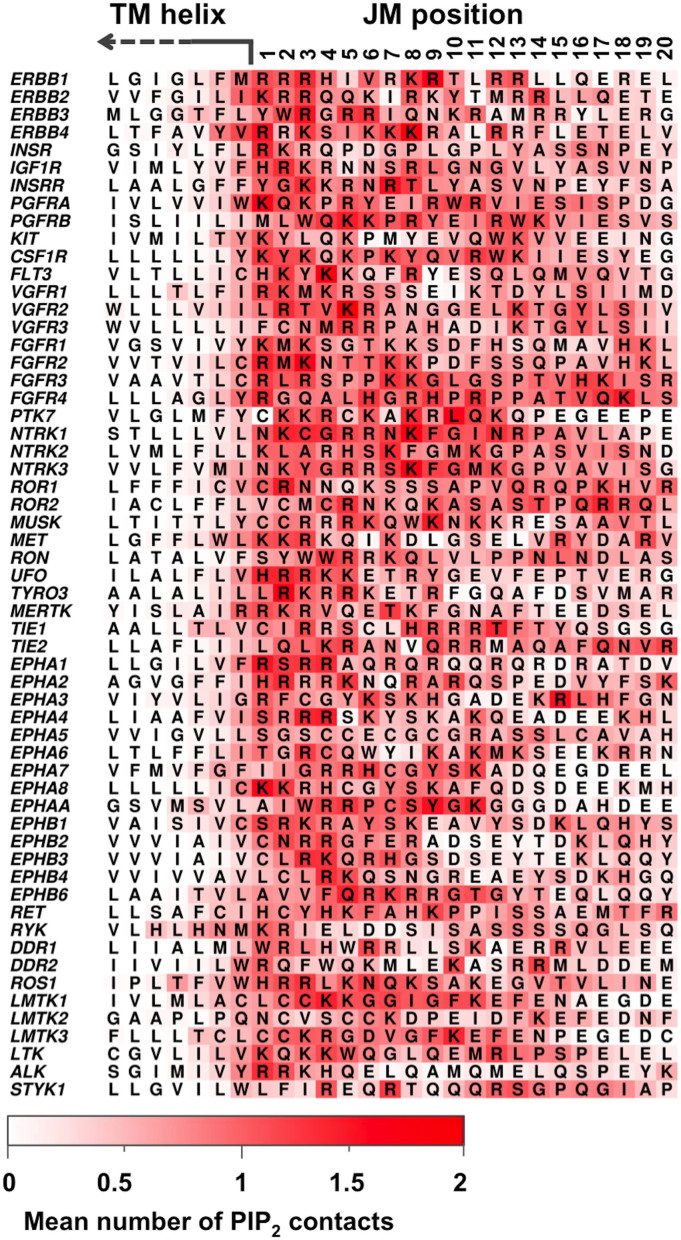
PIP_2_ interactions in all 58 human RTKs. Sequence of the TM-JM region of all 58 RTKs, with each protein residue coloured by the mean number of contacts it formed the phosphoryl group attached to the glycerol moiety of the PIP_2_ headgroup per frame. Contacts were calculated over 3 × 1 μs CG simulation repeats for each RTK. A 6 Å cutoff was used to define contact between the two groups.

Contacts between each residue of the protein and different lipid headgroups were mapped onto the protein structure (Fig. 5) and onto the amino acid sequences (Fig. 6). The overall trend in contacts between the JM region and lipids was in agreement with the findings from our more detailed analysis of the INSR and the EphA2 receptors. Arginine and lysine residues showed a strikingly high level of contact with the PIP_2_ lipid headgroup, with polar residues also showing an overall intermediate level of contact. Similar patterns of contact were observed for PS (Supplementary Fig. 11), but not for PC (Supplementary Fig. 12). PIP_2_ was seen to form the highest overall levels of contact with the JM residues, followed by PS, and then PC. This agrees with RDF and density map data from the INSR and EphA2 receptor systems, showing PIP_2_ has the highest propensity to be distributed around the JM region, followed by PS.

### Correlation between all systems

**Figure 7:**
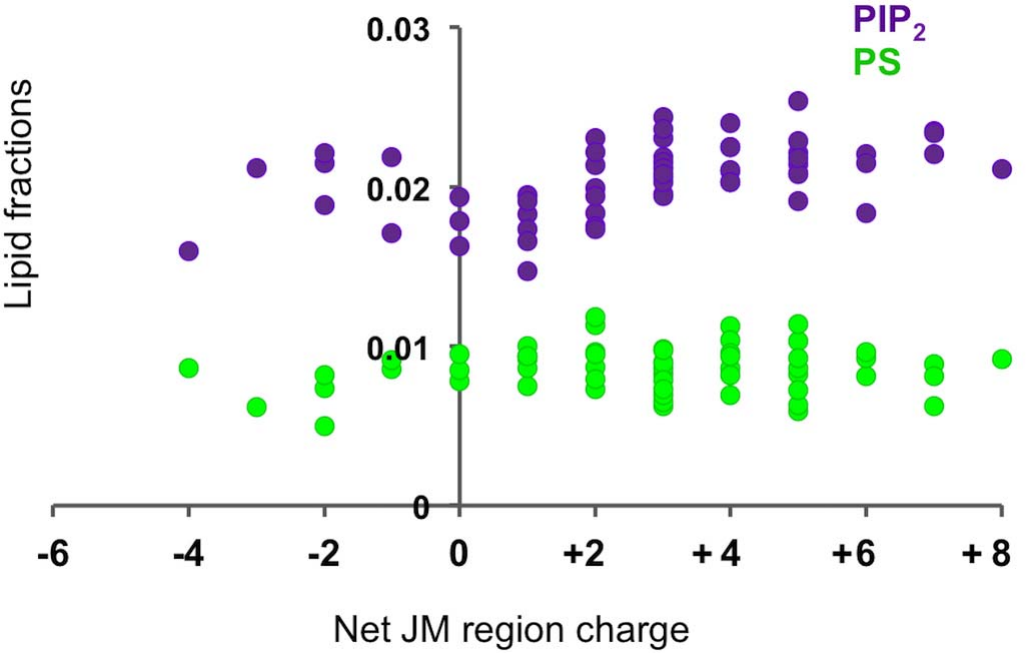
Correlation between JM charge density and PIP_2_ interactions in all 58 human RTKs. Relationship between the relative propensity of PS and PIP_2_ lipid species to cluster around the JM, and net JM region charge for all 58 RTKs. The clustering propensity is measured by computing the mean fraction of each lipid type within 6 Å of the protein per frame, and normalised by dividing by the percentage of that lipid in the inner leaflet.

In all systems we found the same patterns of lipid distribution around the JM region as we saw for the INSR and the EphA2 receptors, with PIP_2_ exhibiting the greatest propensity to cluster around the JM region, followed by PS, and then PC. For those RTKs whose JM region exhibits a net positive charge, a degree of correlation between the net charge and the degree of lipid clustering was observed (Fig. 7). However the degree of clustering seems to saturate at around a net charge of +5. Multiple other factors including charge distribution, JM dynamics, and relative numbers of polar and hydrophobic residues will also influence the degree of lipid clustering and it is therefore not unexpected that a perfect correlation is not observed. Charge distribution is likely to be particularly important; a JM region with all the basic residues grouped at one end will physically be able to accommodate fewer lipids within 6 Å of the charged residues than one in which the same number of charged residues are evenly distributed along the JM region.

### Global analysis of basic residue position and lipid interactions

**Figure 8:**
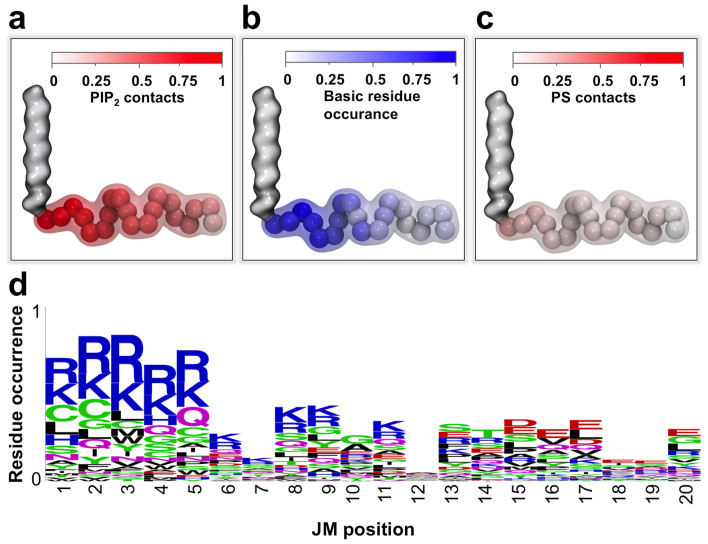
Interactions with PIP_2_ are mainly found in N-terminal end of the JM region. (a) Mean number of PIP_2_ contacts for all 58 human RTKs. TM-JM models coloured by the degree of lipid contact, using contact data from all 58 RTKs. The number of contacts per frame at each JM residue position was summed over all receptors and divided by 58 to give the mean number of contacts per frame at each JM residue position. (b) A hypothetical TM-JM model coloured by the probability of basic residue occurrence at each position of the JM based on all 58 human RTKs. (c) Mean number of PS contacts for all RTKs was calculated as described for (a). (d) Residue occurrence shown as a function of position within the JM region.

We analysed all 58 human RTK simulations more globally. The mean number of lipid contacts formed at each JM residue position over all RTKs was mapped onto a single ‘average' JM structure (Fig. 8). Our results reveal that the overall pattern of PIP_2_ lipid interaction is conserved across the entire RTK family. The highest level of contact with the PIP_2_ lipid headgroup tends to occur at the beginning of the JM region, adjacent to the TM helix (Fig. 8a). Comparison of all 58 RTKs also revealed the highest probability of arginine and lysine occurrence is found within the first five residues of the JM region (Fig. 8b), corresponding to the positions with the highest levels of PIP_2_ lipid headgroup contact. We did not observe a significant enrichment in the mean number of PS lipids within any particular region (Fig. 8c). Our data clearly illustrate the selectivity and specificity of JM-PIP_2_ interactions, and since the same pattern is not observed for PS, the observed PIP_2_ interaction sites are unlikely to be just an effect of the increased membrane surface proximity of this early JM region. The high occurrence of basic residues within the first five residues of the JM region is additionally the overall most conserved sequence feature when considering all 20 residues within all 58 human RTKs simulated here (Fig. 8d).

## Discussion

The present study illustrates the nanoscale lateral organisation of lipids in bilayers around the JM region of the entire human RTK family, both in relatively simple and more complex lipid bilayers. Previous investigations of RTK-lipid interactions have been limited to single species of RTK, and in particular EGFR. The present study extends over the entire family of 58 human RTKs and thus provides novel and more general insights into the molecular details of these interactions and their conserved nature across an *entire family* of receptor proteins. Our simulations reveal that models of the TM helix and a short 20 residue JM regions of all 58 known human RTKs are able to induce bilayer reorganizations in the form of clustering of PIP_2_ and PS lipid molecules into shells around the JM region. Selectivity for certain lipid species was evident, with PIP_2_ found to have the greatest propensity to cluster around the JM regions of all receptors. The observed patterns of lipid distribution agreed with studies of the gp130 cytokine receptor^34^ and dimers of ErbB1^29^, as well as being corroborated by atomistic simulations of full-length ErbB1 which showed PS clustering around the full-length JM regions^15^, and by experimental studies of ErbB1 peptide binding to PIP_2_ monitored by FRET and SPR^27^,^30^. In bilayers with only PS and PC within the inner leaflet, we found that the degree of PS clustering was comparable to that of PIP_2_ in systems containing all three lipid species, suggesting that when present PIP_2_ is able to compete with PS for interaction with the protein. This highlights the importance of lipid diversity in bilayers both *in vivo* and *in silico*, and has implications for the physiological relevance of studies in oversimplified model bilayers. We tested the stability of interactions within more complex lipid membranes, and the same qualitative patterns of PIP_2_ clustering and lipid-protein interaction were observed.

Analysis of contacts formed between different lipid molecules and protein residues over the course of the simulations demonstrated conserved patterns of lipid interaction over all 58 human RTKs, revealing that that these specific interactions were mainly located within the N-terminal part of the JM region. Anionic lipids, and in particular PIP_2_, were seen to form the highest degree of contact with basic residues, an intermediate level of contact with polar residues, and a low level of contact with hydrophobic and acidic residues. This suggests electrostatic interactions between the anionic headgroup moieties of PIP_2_ and PS and clusters of basic residues within the early JM region are the predominant drivers of the observed clustering patterns, with the greater propensity for PIP_2_ to interact with the JM region being a function of its significantly more negative headgroup. In agreement with this observation, *in silico* mutation of basic residues within the JM region of EphA2 receptor to leucines caused a decrease in PIP_2_ and PS clustering and loss of stable contacts at mutated positions. These qualitative conclusions agree with *in vitro* mutagenesis^27^ and computational^29^ studies of ErbB1 in which R/K > N neutralisation mutants were seen to abolish PIP_2_ binding, as well other simulation studies of ErbB1 and the gp130 cytokine receptor which also found high levels of contact between basic JM residues and anionic lipids^15^,^34^.

The results we present here reveal the ability of the short membrane-proximal JM regions of all 58 human RTKs to induce local bilayer reorganizations and clustering of anionic lipids in the inner leaflet around the embedded transmembrane receptors. This behaviour is primarily a result of electrostatic interactions that are localised to a cluster of conserved basic residues found within the first five positions of the JM region, and anionic lipid head groups. The insights that these simulations provide are of interest both for understanding the overall nanoscale organization of proteins and lipids within cell membranes, as well as in terms of RTK function. PIP_2_ is a major modulator of membrane protein activity. For example, it is known to modulate ErbB1 activity^27^, and also to regulate the behaviour of psychostimulant drugs *via* interactions with the dopamine transporter^46^. Thus, the apparent ability of all RTKs to induce clustering of PIP_2_ lipid molecules around their JM regions *via* interactions with conserved residues may therefore have important implications for their regulation. Additionally, PIP_2_ is a central downstream signalling component in a number of cascades controlled by receptor tyrosine kinases, including the PI3K-PKB and PLC-PKC cascades. It has indeed been shown experimentally and computationally that PIP_2_ can form microdomains as a result of protein-lipid interactions^47^ and experimentally that the signalling pathways of RTKs such as EphA2 receptors can be regulated by the surrounding environment^48^,^49^. We can speculate that localization of PIP_2_ around the JM regions of RTKs may therefore be beneficial to the enhancement of signal transduction efficiency within these signalling systems, especially when it is considered that a number of RTKs form higher order arrays at the cell surface^11^,^12^, which could potentially result in an substantial local pool of PIP_2_. Furthermore, the conservation of the physiochemical properties of residues of the N-terminal section of the JM across the entire family of human RTKs and of the resulting lipid interaction hotspots are strongly suggestive of their importance in receptor function, not only on a single protein level but from a broader evolutionary perspective within cellular function.

## Methods

Model proteins consisting of the predicted transmembrane helix-JM region of each receptor tyrosine kinase were built using PyMOL (PyMOL Molecular Graphics System, Version 1.5.0.4 Schrödinger, LLC). The protein sequence was obtained from UniProt^50^ with the predicted transmembrane regions further supported using TMpred^51^. We tested the possible influence of the N-terminal region prior to the TM helix by inclusion of +4 residues at the N-terminus of the INSR TM-JM model, but found no significant impact the predicted on JM-lipid interactions (Supplementary Fig. 13). In the interest of simplicity the N-terminal region was therefore omitted from the protein models used in our simulations. PSIPRED^52^, YASPIN^53^, and JPRED^54^ were used to predict secondary structure within the JM region. Secondary structure was then modelled when agreement was found between two or more prediction programs. The JM regions of each RTK were modelled onto the transmembrane region in an extended conformation to prevent bias and encourage successful self-assembly of the lipid bilayer around the transmembrane helix. This modelling of JM tails was achieved using the sculpting tool in PyMOL and using the command line to set backbone (φ, ψ) angles.

### Coarse-grain molecular dynamics (CG-MD) simulations

All simulations were performed using GROMACS 4.6^55^. Most simulations were performed in membranes containing phosphatidylcholine (PC), phosphatidylserine (PS), and phosphatidylinositol-4,5-bisphosphate (PIP_2_). Additionally, some simulations were performed in more complex membranes containing PC, PS, PIP_2_, GM3, phosphatidylethanolamine (PE), and cholesterol. Atomistic (AT) structures were energy minimized and converted to coarse-grained (CG) representations using the martinize.py workflow, and the MARTINI 2.2 force field was applied with periodic boundary conditions^56^,^57^. A timestep of 20 fs was used for all simulations with Coulomb and van der Waals interactions modelled with a cutoff of 10 Å. Temperature was controlled at 323 K for self-assembly simulations and at 310 K for production run simulations using a Berendsen thermostat^58^ with a coupling constant of 1 ps. Pressure was controlled at 1 bar using a Berendsen barostat with a 1 ps coupling constant. The LINCS algorithm was used to constrain covalent bonds to their equilibrium values^59^. The proteins were inserted into a POPC bilayer using self-assembly simulations^37^ for 25 ns with a time step of 20 fs as previously described for the gp130 receptor^34^. The simulations were run with the standard MARTINI water^57^ and then neutralised and ionized using 0.15 M NaCl. The membrane composition was subsequently changed into the desired composition utilizing an exchange lipids approach previously published^42^. The script randomly exchanges POPC with specified lipids in each leaflet before re-solvating, re-ionizing and energy minimizing the system. The production runs of the TM-JM models embedded in asymmetric bilayers were simulated for 1 μs of CG MD simulation at 310 K. Each 1 μs production run was repeated three times with different initial velocity seeds and with different random position of the lipids.

### Atomistic molecular dynamics (AT-MD) simulations

For INSR and the EphA2 receptor TM-JM region systems, a CG to AT fragment based protocol^60^ was used to convert the final frame of each 1 μs CG repeat to AT detail. Simulations were performed using the united-atom GROMOS 53a6^61^ force field in GROMACS 4.6^55^. Electrostatics were modelled using the Particle Mesh Ewald (PME) model^62^ with a cutoff of 10 Å, while a cutoff of 10 Å was also used for van der Waals interactions. Temperature was controlled at 310 K by a V-scale thermostat^63^ and pressure controlled at 1 bar utilizing the Parinello-Rahman barostat^64^. The LINCS algorithm was used to constrain covalent bonds to their equilibrium values^59^.

After conversion to AT detail each system was re-solvated, re-ionized and energy minimized before being equilibrated for 1 ns with position restraints applied to the Cα atoms of the protein with a force constant of 10 kJ/mol/Å^2^. Each AT simulation was then run for 50 ns with a 2 fs time step.

### Analysis

VMD plugins were used to compute radial distribution functions and generate VolMap density surfaces^65^,^66^. The RDF data was normalised such that the area under the curve for each lipid species was equal to one to enable ready comparison between different lipid species. Contact data was mapped onto sequence using ALINE^67^, whilst mapping of contact data onto structure was achieved utilizing the β-factor columns of .pdb structure files, and visualised using VMD^66^. Further analysis was carried out through VMD using locally developed scripts. Figure 8d was generated utilizing the WebLogo server^68^.

## Author Contributions

G.H. and H.K. performed all the experiments. G.H. and H.K. performed the data analyses. M.S.P.S. and H.K. designed the experiments. G.H., M.S.P.S. and H.K. wrote the article.

## Supplementary Material

Supplementary Information Supplementary Information
