# Supplementary material for: The juxtamembrane regions of human receptor tyrosine kinases exhibit conserved interaction sites with anionic lipids
Source: Sci Rep. 2015 Mar 17;5:9198. doi: 10.1038/srep09198 (PMC4361843; doi:10.1038/srep09198)
Supplement: Supplementary Information [file srep09198-s1.pdf]

# Supplementary Information

# The juxtamembrane regions of human receptor tyrosine kinases exhibit conserved interaction sites with anionic lipids

*George Hedger, Mark S. P. Sansom and Heidi Koldsø*[1]

Department of Biochemistry, University of Oxford, South Parks Road, Oxford OX1 3QU, United Kingdom

## Supplementary Figures

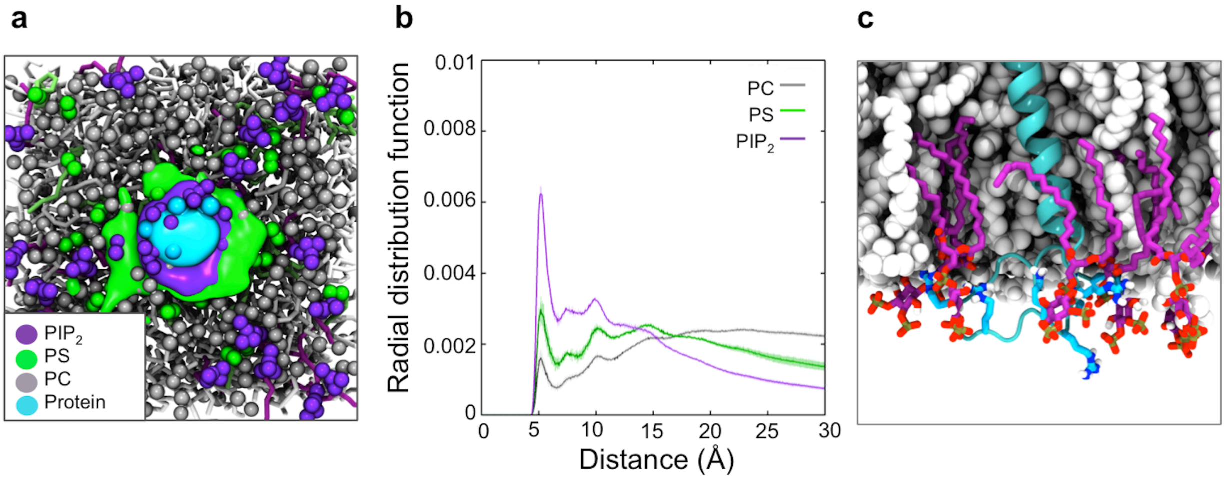


**Supplementary Figure 1 | Lipids clustering around the JM region within the EphA2 system** Lipids clustering around the JM region within the EphA2 receptor system. (**a**) Occupancy density maps of the inner leaflet surface showing spatial occupancy for the phosphoryl group attached to the glycerol moiety of the $PIP_2$ head group (purple), the PS head group (green), and the Arg and Lys residues of the JM (cyan). Occupancy maps were computed from concatenated 3 x 1 µs simulations of each system, using the Volmap VMD. (**b**) Radial distribution functions (RDFs) for lipid head groups of $PIP_2$, PS, and PC with respect to the EphA2 receptor. The RDFs shown are the mean values from 3 x 1 µs simulation repeats, with the standard deviation displayed as error bars. (**c**) A cluster of $PIP_2$ lipids around the EphA2 receptor JM region. The protein has been shown in cyan with the basic juxtamembrane regions in sticks coloured according to atom type with carbon atoms in cyan. $PIP_2$ lipids within 6 Å of the protein have been shown as sticks coloured according to atom type, with carbon atoms in purple. The PC and PS lipids are shown as gray spheres.

[1] Correspondence may be addressed to heidi.koldsoe@bioch.ox.ac.uk

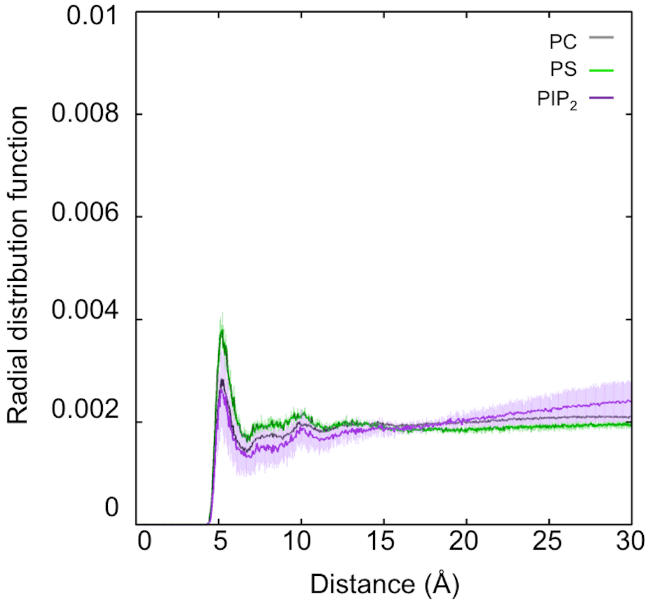


**Supplementary Figure 2 | Lipid organization in TM only system.** Radial distribution function (RDF) for the $PIP_2$ (purple), PS (green), and PC (grey) lipid head groups relative to the control system of the INSR transmembrane helix without any JM region attached. The RDFs of each lipid species show reduced magnitude and no significant difference between different lipid species as compared to INSR with JM region attached (Fig 3b). All RDFs were computed over 3 x 1 µs CG repeats of each system with random initial velocity seeds, with the standard deviation indicated as error bars.

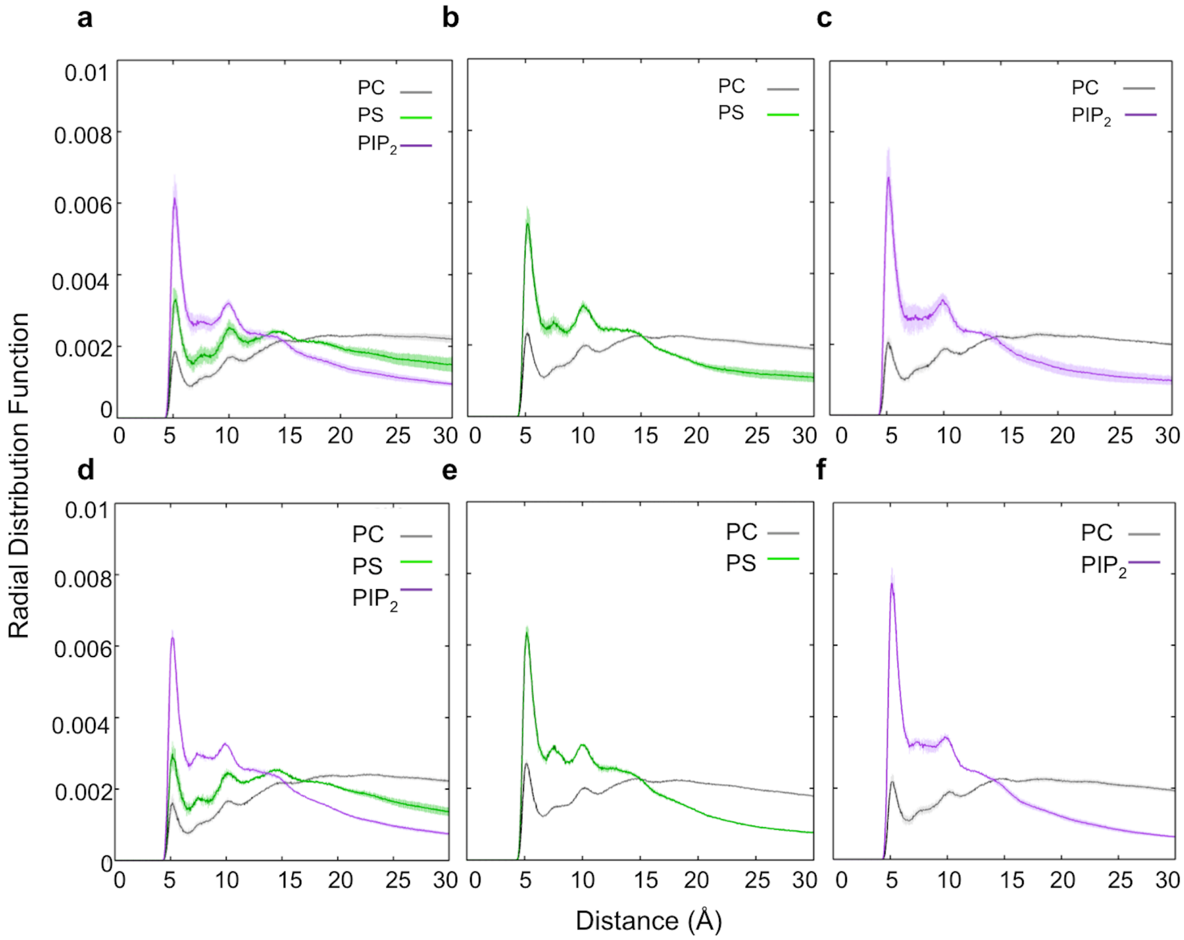


**Supplementary Figure 3 | Lipid organization in different membrane environments for the INSR and EphA2 systems.** Radial distribution function plots for the PC, PS, and $PIP_2$ lipid head groups within systems containing INSR and EphA2 receptor embedded in bilayers of different inner leaflet composition. The RDFs shown are the mean values from 3 x 1 µs simulation repeats, with the standard deviation displayed as error bars. (**a**) INSR system with an inner leaflet composition of PC:PS:$PIP_2$ (**b**) INSR system with an inner leaflet composition of PC:PS (**c**) INSR system with an inner leaflet composition of PC:$PIP_2$. (**d**) EphA2 receptor system with an inner leaflet composition of PC:PS:$PIP_2$ (**e**) EphA2 receptor system with an inner leaflet composition of PC:PS (**f**) EphA2 receptor system with an inner leaflet composition of PC:$PIP_2$.

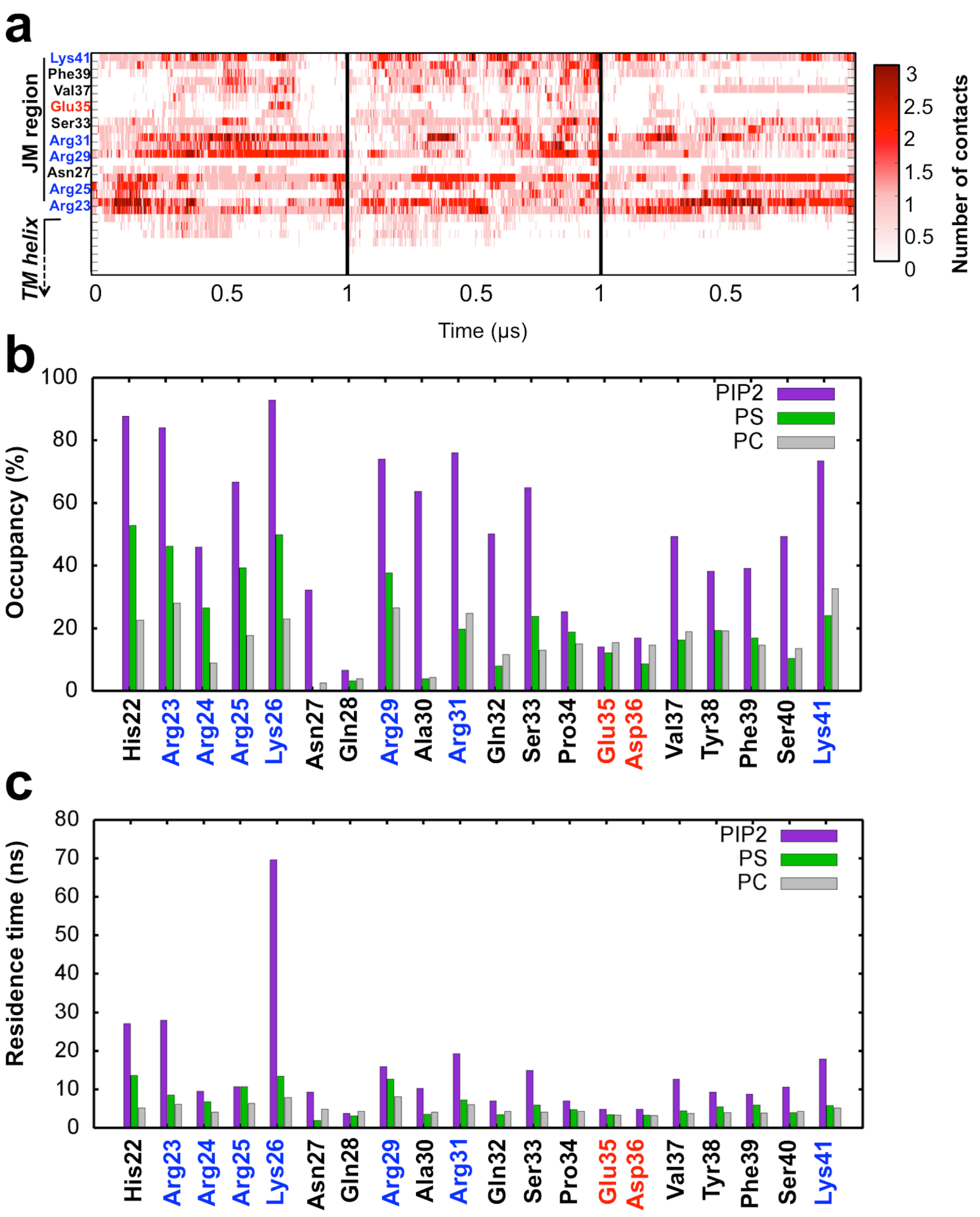


**Supplementary Figure 4 | Dynamics of $PIP_2$ interaction with the JM region in EphA2 receptor simulations. (a)** Matrix shows the number of contacts per frame between each residue of the EphA2 receptor JM region and the $PIP_2$ lipid head group as a function of time. The time course shown is from concatenated 3 x 1 μs CG simulation repeats and a cutoff of 6 Å was used to define contact. **(b)** Percentage of simulation time at least one lipid is in contact with each residue within the JM region. $PIP_2$, PS and PC are shown in purple, green and gray respectively. (**c**) Mean residence time (occupancy time divided by number of association events) for 3 runs plotted for each JM residues for $PIP_2$, PS and PC.

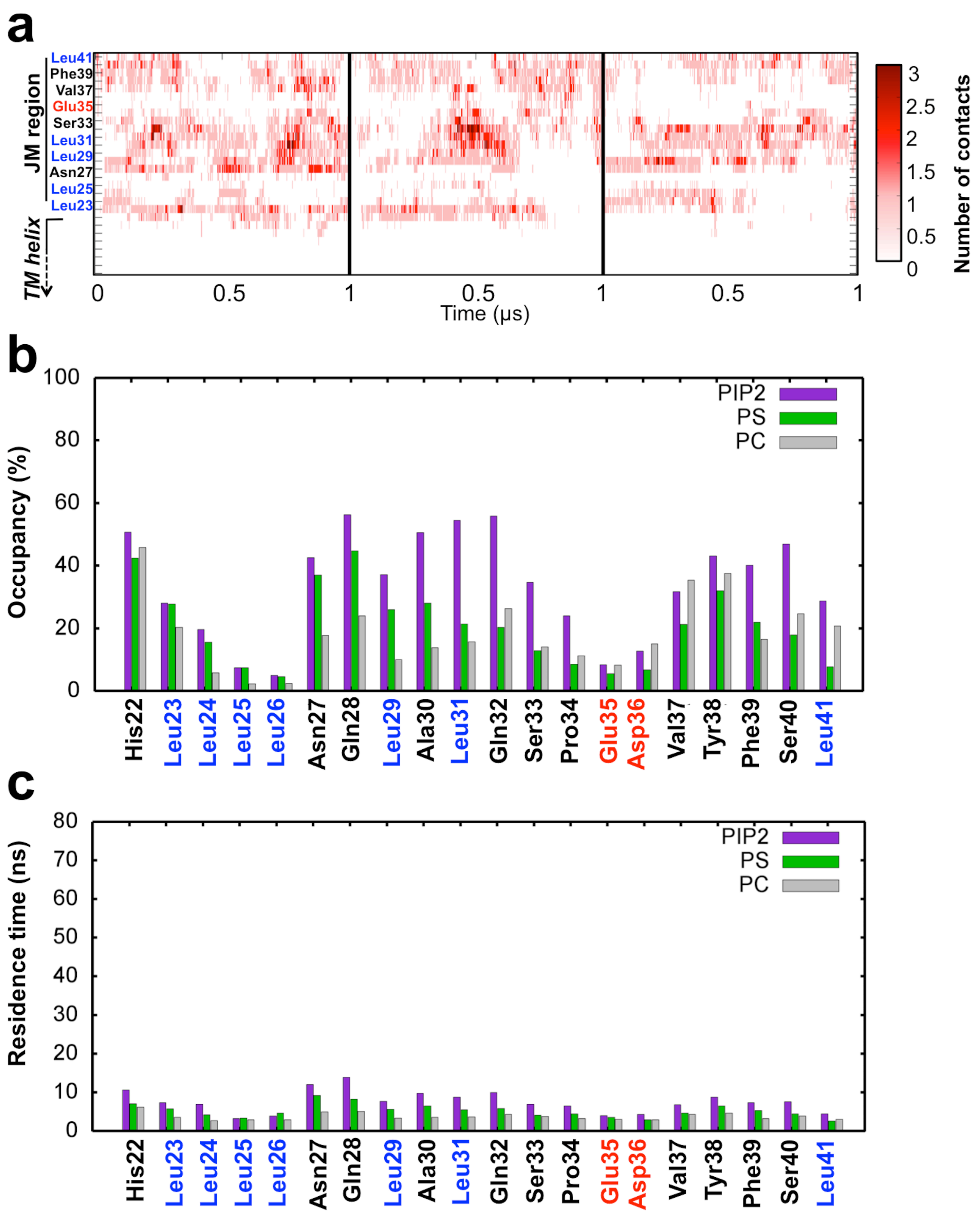


**Supplementary Figure 5 | Dynamics of $PIP_2$ interaction with the JM region in mutant EphA2 receptor simulations. (a)** Matrix shows the number of contacts per frame between each residue of the EphA2 receptor JM region and the $PIP_2$ lipid head group as a function of time. The time course shown is from concatenated 3 x 1 μs CG simulation repeats and a cutoff of 6 Å was used to define contact. **(b)** Percentage of simulation time at least one lipid is in contact with each residue within the JM region. $PIP_2$, PS and PC are shown in purple, green and gray respectively. (**c**) Mean relative residence time (occupancy time divided by number of association events) for 3 runs plotted for each JM residues for $PIP_2$, PS and PC.

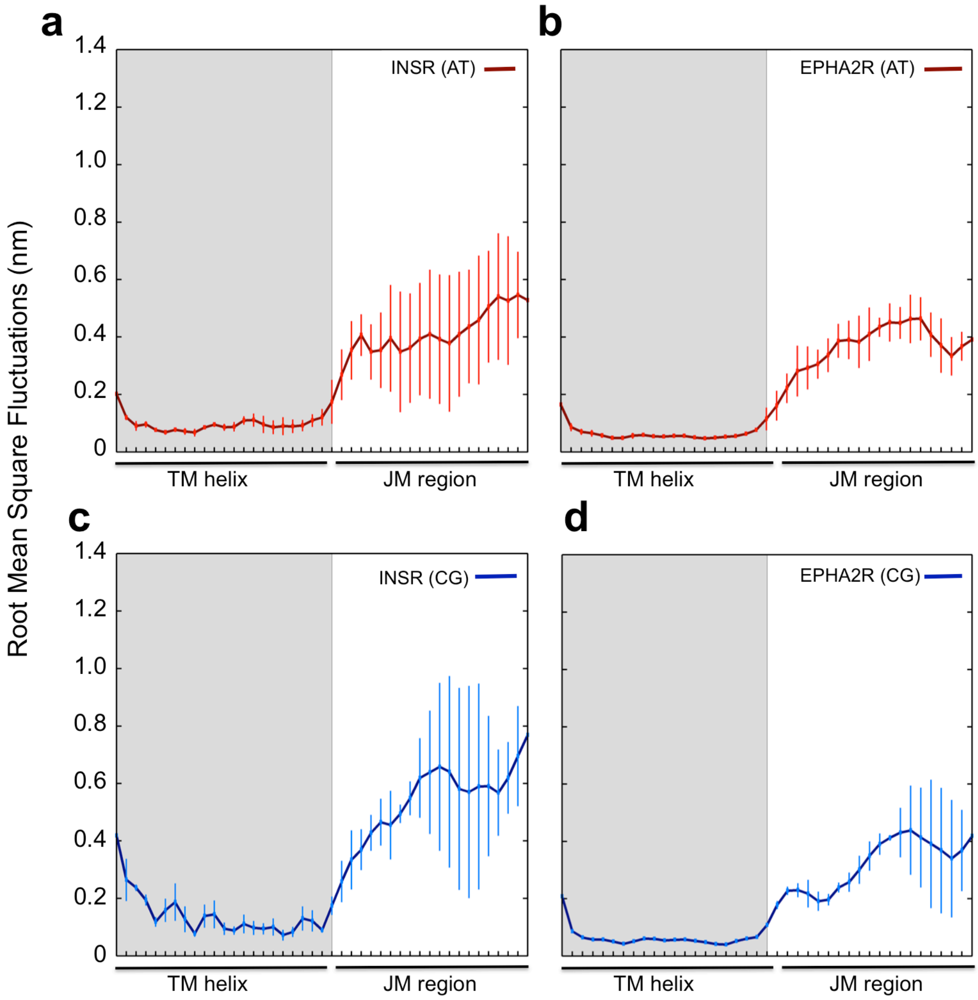


**Supplementary Figure 6 | Root mean square fluctuations for the INSR and EphA2 receptor systems in CG and AT detail.** Each simulation was centered onto the TM helix and automatically fitted by the g_rmsf function of GROMACS, before RMSF values were computed for each backbone bead in the CG simulations, and each Cα bead in the AT simulations. Only the last 50 ns of each CG simulation were used in these calculations to allow better comparison with AT systems. The data has been plotted as the mean value from three simulation repeats, with the standard deviation indicated as error bars. (**a**) RMSF from atomistic simulation of the INSR system. (**b**) RMSF from atomistic simulation of the EphA2 receptor system. (**c**) RMSF from CG simulation of the INSR system. (**d**) RMSF from CG simulation of the EphA2 receptor system

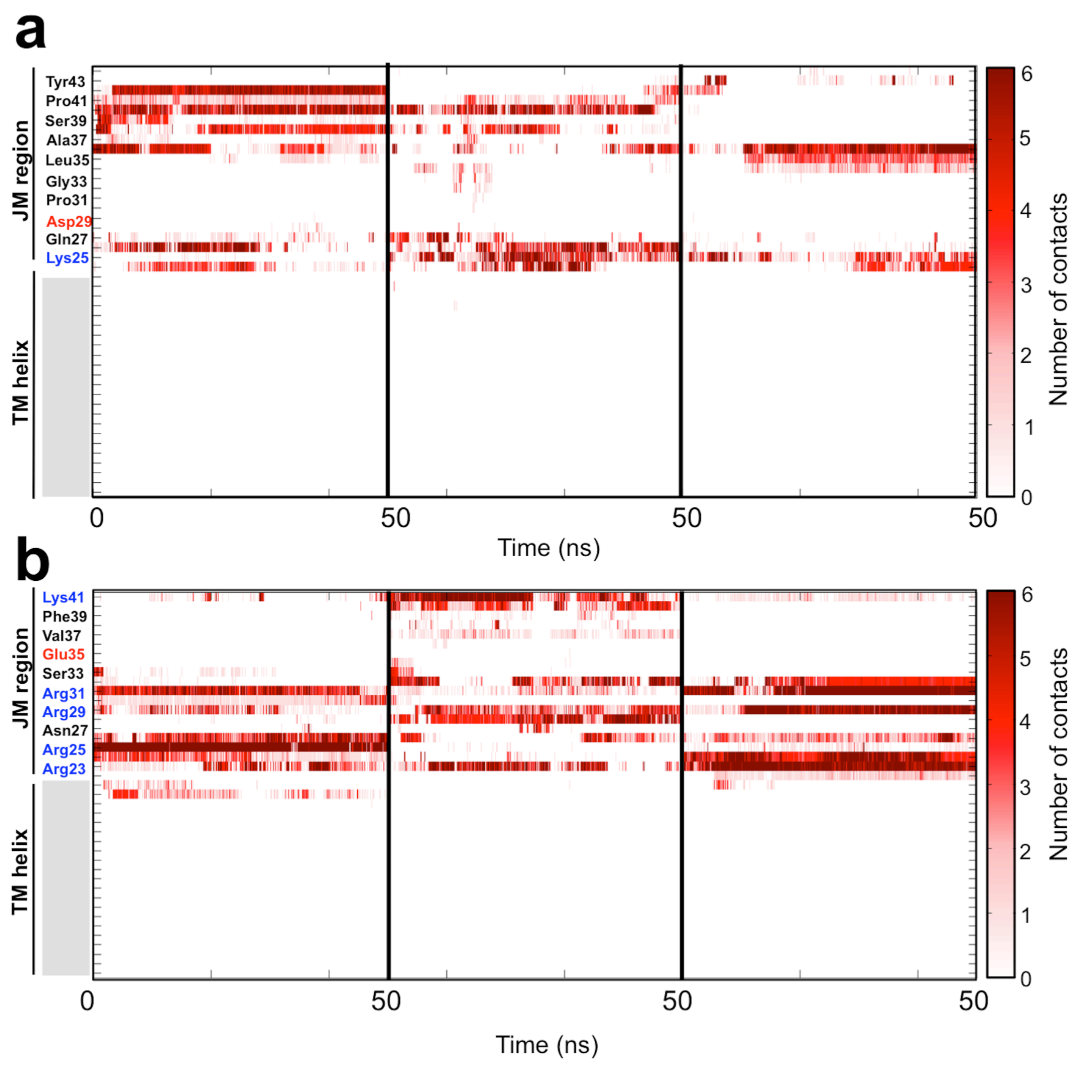


**Supplementary Figure 7 | Interactions over time between protein and $PIP_2$ within the atomistic simulations.** The time course shown is from concatenated 3 x 50 ns AT simulation repeats. A distance cutoff of 4 Å was used to define contact. (**a**) INSR AT simulation contact over time matrices between each JM residue and the lipid head groups of $PIP_2$. (**b**) EphA2 receptor AT simulation contact over time matrices between each JM residue and the lipid head groups of $PIP_2$.

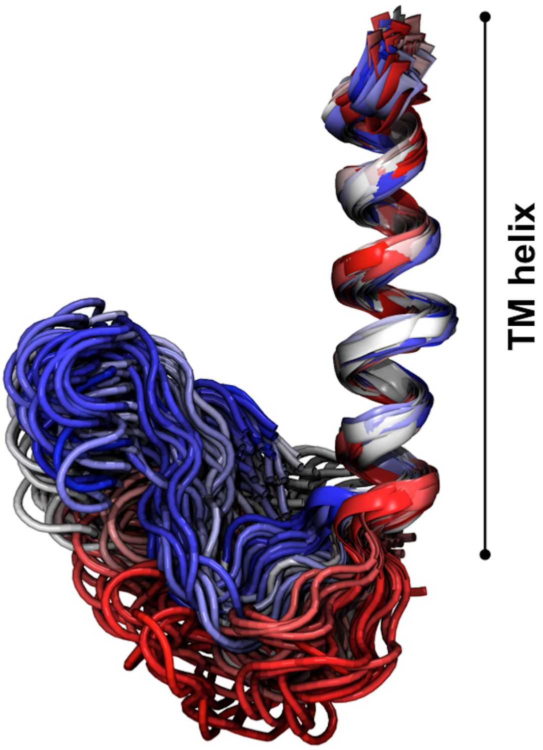


**Supplementary Figure 8 | Dynamics of the TM-JM model of INSR.** 100 evenly distributed structures are shown over the course of one 50 ns production run, and coloured by timestep (from red to blue). Each structure is aligned on the TM helix

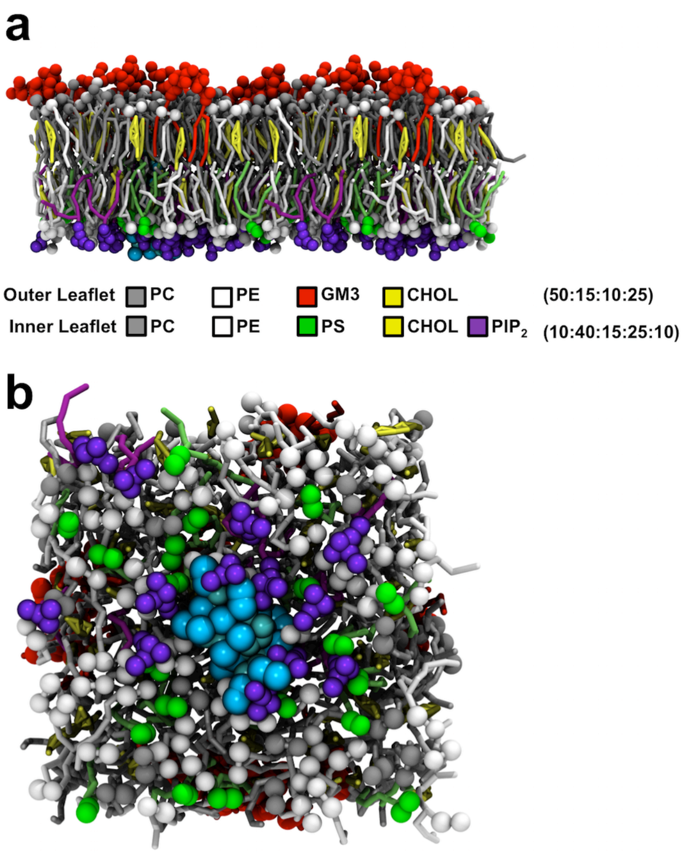


**Supplementary Figure 9 | $PIP_2$ clustering is also observed in complex physiologically relevant membranes.** A model physiological bilayer with embedded INSR protein model (cyan). The composition of the outer leaflet is PC:PE:GM3:CHOL (50:15:10:25) and the lower leaflet is PC:PE:PS:$PIP_2$:CHOL (10:40:15:10:25). (**a**) side on cross-section, (**b**) the inner leaflet surface. $PIP_2$ lipids (purple) are clustering around the protein (cyan)

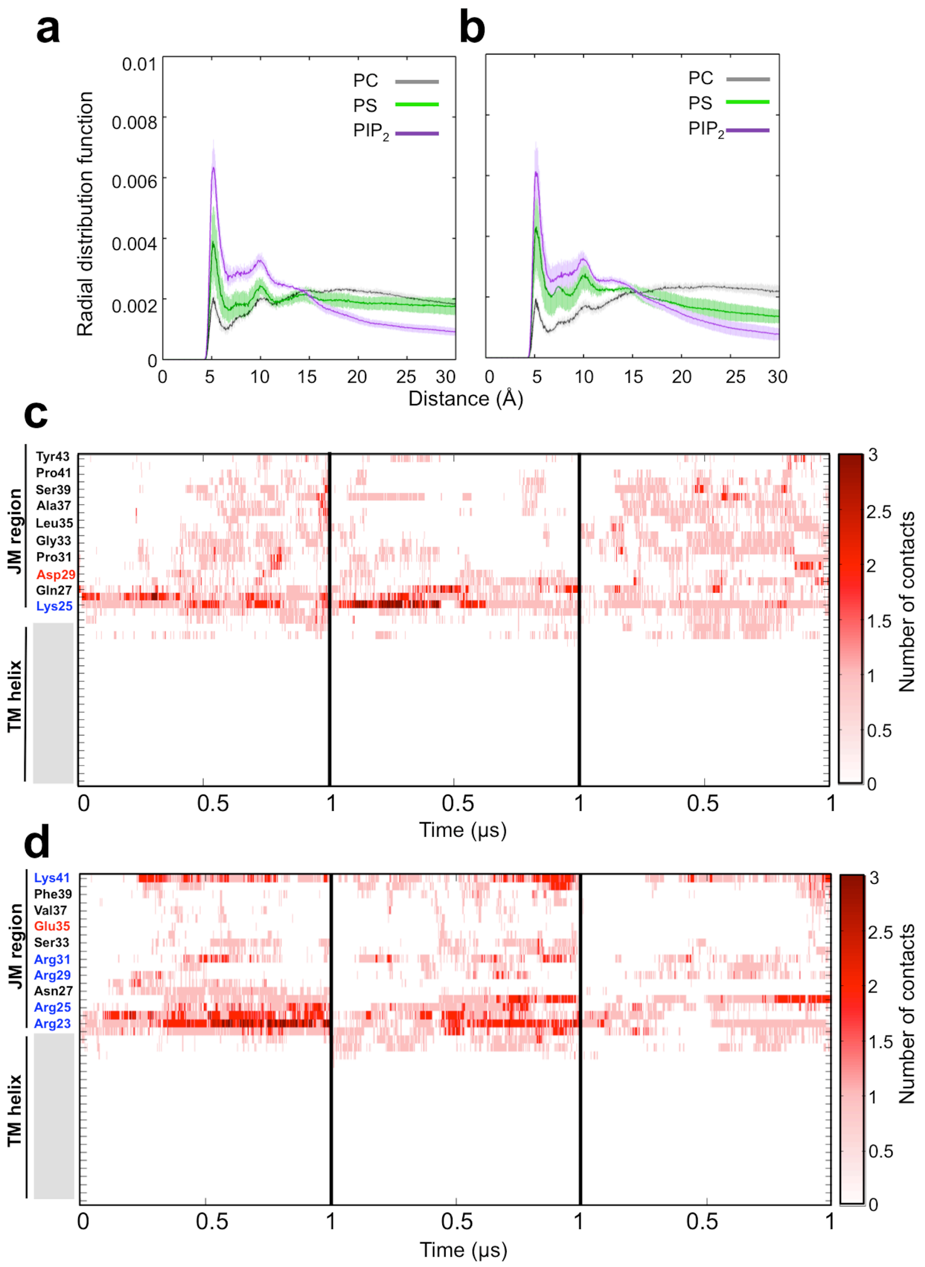


**Supplementary Figure 10 | Lipid organization and PIP$_2$ clustering in complex physiologically relevant membranes.** (**a**)+(**b**)RDFs for the lipid head groups of PIP$_2$ (purple), PS (green) and PC (grey) in physiological membranes, relative to the protein backbone. The standard deviation is shown as error bars, and is comparatively larger than for simple bilayers due to the smaller number of PIP$_2$, PS and PC molecules. These RDFs were computed over 3 x 1 µs CG repeats for each system, with different initial velocity. (**a**) The INSR system and (**b**) the EphA2 receptor system (**c**)+(**d**) Interactions between protein residues and PIP$_2$ over time are displayed for (**c**) the INSR embedded in a complex membrane environment and (**d**) the EphA2 receptor embedded in a complex membrane environment. Basic residues are highlighted in blue and acidic residues in red. The time course shown is from concatenated 3 x 1 µs CG simulation repeats and a cutoff of 6 Å was used to define a contact.

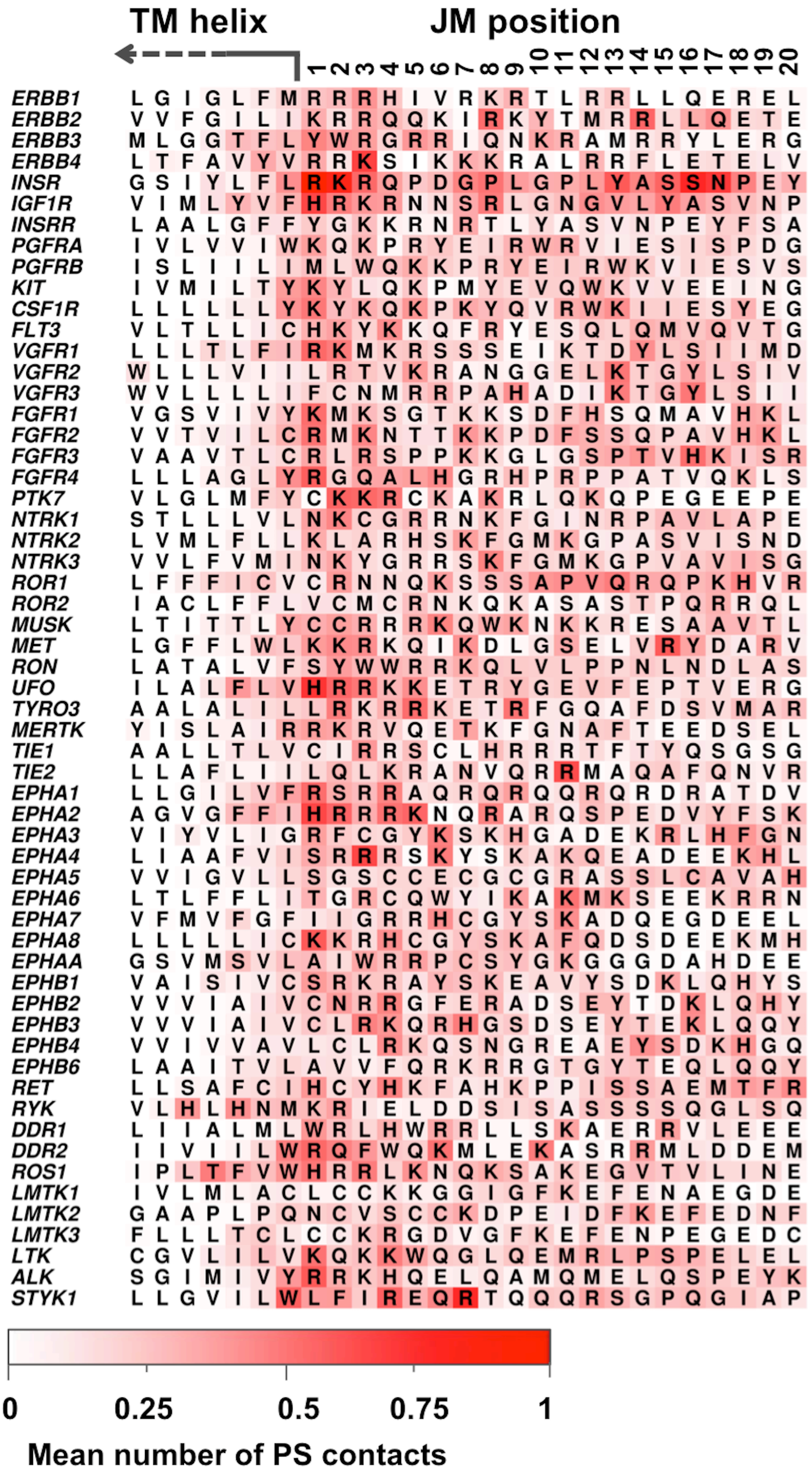


**Supplementary Figure 11 | PS interactions in all 58 human RTKs.** Sequence of the TM-JM region of all 58 RTKs, with each protein residue coloured by the mean number of contacts it formed the phosphoryl group attached to the glycerol moiety of PS head group per frame. Contacts were calculated over 3 x 1 µs CG simulation repeats for each RTK. A 6 Å cutoff was used to define contact between two selections..

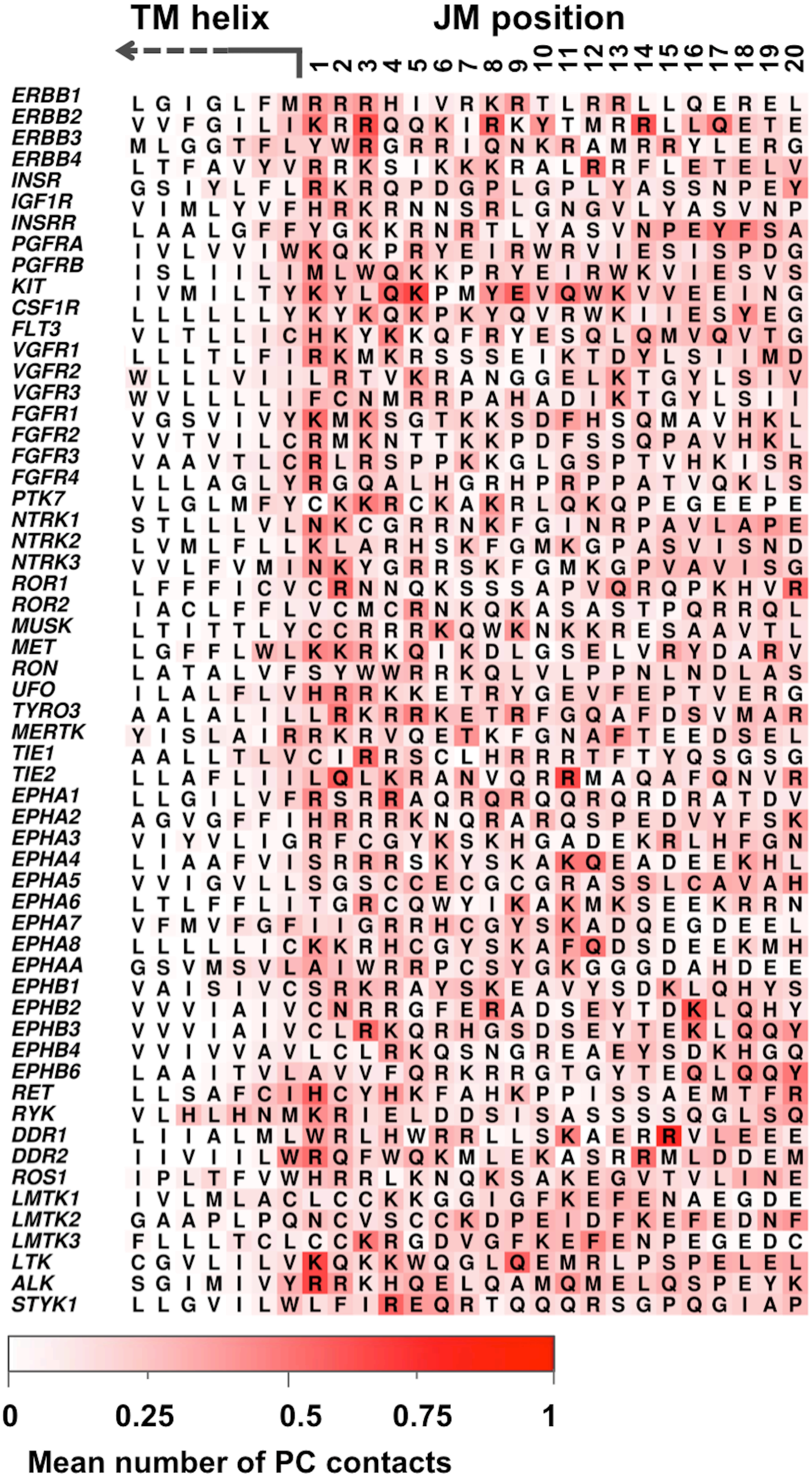


**Supplementary Figure 12 | PC interactions in all 58 human RTKs.** Sequence of the TM-JM region of all 58 RTKs, with each protein residue coloured by the mean number of contacts it formed the phosphoryl group attached to the glycerol moiety of PC head group per frame. Contacts were calculated over 3 x 1 µs CG simulation repeats for each RTK. A 6 Å cutoff was used to define contact between two selections.

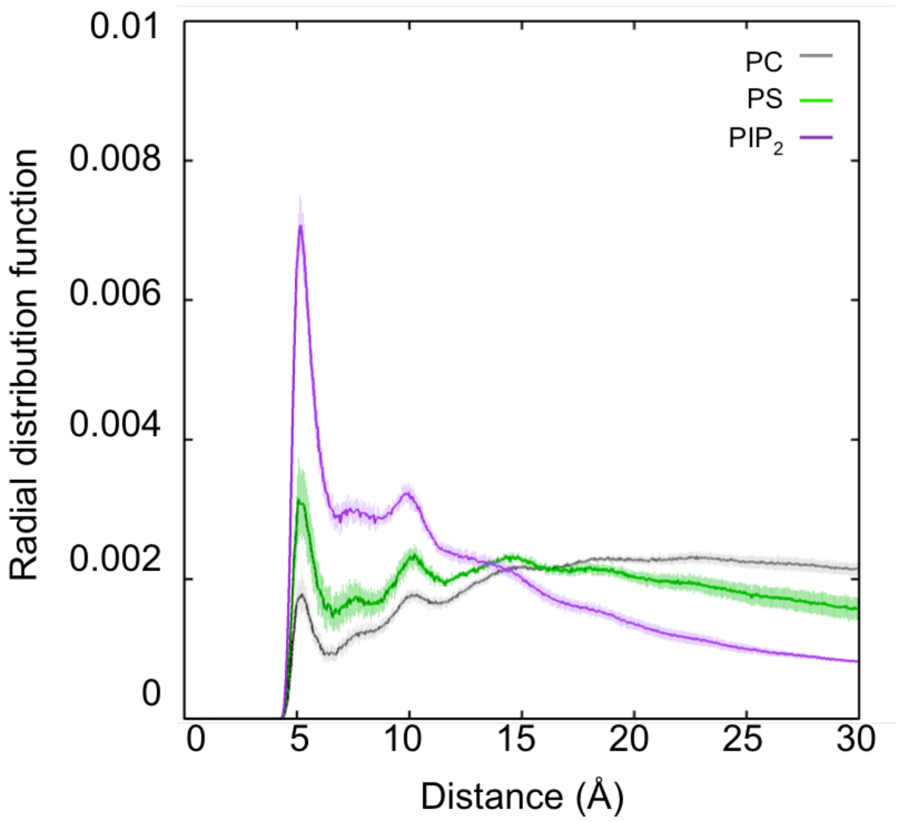


**Supplementary Figure 13 | Lipid organization in the INSR +4 system.** Radial distribution function (RDF) for the $PIP_2$ (purple), PS (green), and PC (grey) lipid head groups relative to a control system of the INSR TM-JM peptide modelled with an additional +4 residues on the N-terminal side. The relative RDFs of each lipid species shows no significant deviation compared to those observed in the absence of an N-terminal region (Fig 3b). In the interest of simplicity and expediting data collection the N-terminal region was therefore omitted from the protein models used in our simulations. All RDFs were computed over 3 x 1 µs CG repeats of each system with random initial velocity seeds, with the standard deviation indicated as error bars.
